# The Inclusion of Ethnic Minority Patients and the Role of Language in Telehealth Trials for Type 2 Diabetes: A Systematic Review

**DOI:** 10.2196/jmir.6374

**Published:** 2016-09-26

**Authors:** Talia Isaacs, Daniel Hunt, Danielle Ward, Leila Rooshenas, Louisa Edwards

**Affiliations:** ^1^ University of Bristol Graduate School of Education Bristol United Kingdom; ^2^ University of Nottingham School of English Nottingham United Kingdom; ^3^ University of Bristol School of Social Policy Bristol United Kingdom; ^4^ University of Bristol School of Social and Community Medicine Bristol United Kingdom

**Keywords:** telemedicine, telehealth, type 2 diabetes, diabetes mellitus, ethnic minorities, trial recruitment, systematic review, language, English proficiency, health communication

## Abstract

**Background:**

Type 2 diabetes is a serious, pervasive metabolic condition that disproportionately affects ethnic minority patients. Telehealth interventions can facilitate type 2 diabetes monitoring and prevent secondary complications. However, trials designed to test the effectiveness of telehealth interventions may underrecruit or exclude ethnic minority patients, with language a potential barrier to recruitment. The underrepresentation of minorities in trials limits the external validity of the findings for this key patient demographic.

**Objective:**

This systematic review examines (1) the research reporting practices and prevalence of ethnic minority patients included in telehealth randomized controlled trials (RCTs) targeting type 2 diabetes and the trial characteristics associated with recruiting a high proportion of minority patients, and (2) the proportion of included RCTs that report using English language proficiency as a patient screening criterion and how and why they do so.

**Methods:**

Telehealth RCTs published in refereed journals targeting type 2 diabetes as a primary condition for adults in Western majority English-speaking countries were included. Ethnically targeted RCTs were excluded from the main review, but were included in a post hoc subgroup analysis. Abstract and full-text screening, risk of bias assessment, and data extraction were independently conducted by two reviewers.

**Results:**

Of 3358 records identified in the search, 79 articles comprising 58 RCTs were included. Nearly two-thirds of the RCTs (38/58) reported on the ethnic composition of participants, with a median proportion of 23.5% patients (range 0%-97.7%). Fourteen studies (24%) that included at least 30% minority patients were all US-based, predominantly recruited from urban areas, and described the target population as underserved, financially deprived, or uninsured. Eight of these 14 studies (57%) offered intervention materials in a language other than English or employed bilingual staff. Half of all identified RCTs (29/58) included language proficiency as a participant-screening criterion. Language proficiency was operationalized using nonstandardized measures (eg, having sufficient “verbal fluency”), with only three studies providing reasons for excluding patients on language grounds.

**Conclusions:**

There was considerable variability across studies in the inclusion of ethnic minority patients in RCTs, with higher participation rates in countries with legislation to mandate their inclusion (eg, United States) than in those without such legislation (eg, United Kingdom). Less than 25% of the RCTs recruited a sizeable proportion of ethnic minorities, which raises concerns about external validity. The lack of objective measures or common procedures for assessing language proficiency across trials implies that language-related eligibility decisions are often based on trial recruiters’ impressionistic judgments, which could be subject to bias. The variability and inconsistent reporting on ethnicity and other socioeconomic factors in descriptions of research participants could be more specifically emphasized in trial reporting guidelines to promote best practice.

**Trial Registration:**

PROSPERO International Prospective Register of Systematic Reviews: CRD42015024899; http://www.crd.york.ac.uk/PROSPERO/display_record.asp?ID=CRD42015024899 (Archived by WebCite at http://www.webcitation.org/6kQmI2bdF)

## Introduction

Diabetes, a chronic metabolic condition, is on the rise, placing growing resource pressures on health care systems worldwide [1]. With the risk of developing diabetes increasing with obesity, sedentary behavior, and age, type 2 diabetes accounts for roughly 90% of diabetes cases. Behavioral changes (eg, diet, exercise), coupled with medication aiming to lower blood sugar levels and blood pressure, can reduce the risk of developing diabetes-related complications [2]. Due to its prevalence and the benefits of effective disease management, type 2 diabetes is frequently targeted in studies aiming to promote lifestyle modification through behavioral interventions and education [3].

Telehealth, broadly defined as remote health care delivery using technology [4], can partially alleviate the growing pressures associated with aging populations and rising rates of chronic conditions [5]. Technological advances have led to the availability of wide-ranging telehealth options to support patients as an alternative or supplement to traditional outpatient care, with presumed benefits including cost reduction, increasing convenience and access, and promoting patient self-management [6,7]. For example, diabetes-related telehealth services often involve platforms for measuring and communicating blood glucose information and receiving feedback from an automated system or remote professional. Other services involve structured self-management education or peer or motivational support [8]. Although findings from randomized controlled trials (RCTs) testing the effectiveness of telehealth interventions have been mixed and tend to vary by condition, systematic reviews focusing on type 2 diabetes specifically have generally yielded positive results, including modest but significant improvements for glycemic control and favorable outcomes for patient quality of life and treatment satisfaction [8-15].

Due to its flexibility, telehealth has the potential to reach underserved patients who may experience difficulties accessing traditional health services [16,17]. This extends to ethnic minorities, who are particularly vulnerable to developing type 2 diabetes [18], tending to do so at a younger age and with a lower body mass index than the general population in Western countries [19]. In the United Kingdom, for example, South Asians have up to six times higher prevalence of type 2 diabetes compared to people from Caucasian backgrounds, with incidence up to three times higher in people of African or African-Caribbean descent [20]. Ethnic minority groups also experience poorer long-term outcomes for diabetes, such as worse glycemic control and higher rates of complications [21], even when patients have access to health care at minimal cost [22] and after adjusting for age and socioeconomic status [19,23]. Ethnic minorities may also experience impaired self-management and underuse services due to a combination of socioeducational factors, including a less developed understanding of diabetes, differing attitudes toward medication, culturally specific dietary practices, and/or language barriers to accessing care [13,24,25].

Despite having a higher incidence of diabetes, ethnic minority patients tend to be underrepresented in trials [26-28]. This means that they cannot receive the potential health benefits from trial participation [29,30], including access to improved treatments and closer monitoring during the trial period. In addition, studies are not able to adequately assess the effectiveness of new treatments for these higher-need groups. For example, telehealth technologies trialed on a disproportionately Caucasian sample may not be generalizable to a diverse patient demographic, with implications for the service-level adoption of such interventions [31,32]. Low participation rates of ethnic minority diabetes patients have been reported in systematic reviews focusing on telehealth interventions, although the ethnic composition of the recruited sample is often unreported. In a 2006 review, only eight of 26 included studies reported on the ethnic composition of trial participants, of which the median proportion of ethnic minority patients was 39% (range 5%-100%) [33]. Similarly, two 2014 reviews found that only half of 16 included studies [34] and four of nine included studies [3] reported on the ethnic makeup of the recruited sample, with variable minority participation across these studies (range 15%-100% and range 24%-100% of total participant population, respectively).

Although the results of these reviews [3,33,34] are revealing in terms of prevalence and research reporting practices, they have several limitations. First, the included studies were restricted to a narrow range of computer-based telehealth interventions—a subset of the wide variety of the telehealth technologies available. For example, they excluded trials that were phone-based or included glucose monitoring, which are pervasive in telehealth diabetes research [15]. Next, two reviews included studies that targeted both type 1 and type 2 diabetes [33,34]. Because ethnic minorities are not disproportionately affected by type 1 diabetes, it is important to identify the prevalence of minority participants in studies that exclusively focus on type 2 diabetes. Third, two of the reviews included trial designs other than RCTs [3,33], with different designs potentially affecting the type of patients who had opted to take part (eg, due to time commitments) [35,36] in addition to not providing the same design safeguards against bias [37]. Finally, both 2014 reviews included studies that specifically recruited from one or more ethnic minority communities [3,34]. The inclusion of these ethnically targeted studies likely inflated ethnic minority patient prevalence estimates relative to studies recruiting from the general population. This systematic review addresses these limitations by focusing on telehealth RCTs targeting type 2 diabetes in adults recruited from the wider patient population. The telehealth medium used in the RCTs was not restricted in order to more comprehensively survey research reporting practices across the range of technologies. A central aim of this review was to explore the barriers and facilitators to ethnic minority inclusion in telehealth RCTs.

Among the factors that could affect ethnic minority participation in RCTs, language and literacy are often identified as potential obstacles [26,31]. Of foreign-born people living in the United States, 29% reported speaking English “not well” or “not at all” [38] compared to more than 12% in Australia [39]. In England and Wales, nearly 19% of adults from the four largest ethnic minority communities were estimated to speak little or no English [40]. Poor language skills are also related to higher levels of undiagnosed diabetes and to difficulties accessing services [13,41]. Ensuring that patients have the requisite language ability to understand the conditions for trial participation is an ethical imperative in all research (eg, obtaining informed consent). Because communication is a key part of the treatment in telehealth trials, language could act as a barrier to telehealth’s ability to provide more accessible, equitable modalities for delivering care. For example, telehealth interventions often necessitate basic literacy skills (eg, understanding and inputting written text) or enhanced communication skills (eg, communicating on the phone with no access to nonverbal cues), potentially barring the participation of patients without adequate language skills to engage with the intervention in the absence of translation or interpretation services [42].

There is a pressing need to examine the role of language in telehealth interventions, especially in countries where a sizeable portion of the population has limited ability in the official language. This is particularly the case for conditions such as type 2 diabetes, to which ethnic minority communities are particularly vulnerable. Yet little is known about whether and how patients are screened for language proficiency or literacy nor the extent to which these factors are cited among the participant inclusion or exclusion criteria in telehealth RCTs targeting type 2 diabetes [30,43]. In light of these gaps, the goals of this systematic review were to investigate (1) the research reporting practices and prevalence of ethnic minority patients included in telehealth RCTs targeting type 2 diabetes and trial characteristics associated with successful minority patient recruitment, and (2) the proportion of included RCTs that report English language proficiency as a participant-screening criterion and how and why proficiency was assessed.

## Methods

This review followed the Cochrane Collaboration’s handbook on conducting systematic reviews [44] and the Preferred Reporting Items for Systematic Reviews and Meta-Analyses (PRISMA) guidelines [45]. Full methodological details, including MEDLINE search strategy terms, are reported in the published protocol [46].

### Search Strategy and Study Screening and Selection

The search, which was conducted in late August 2015, included studies published from January 1, 2000 to July 31, 2015 using MEDLINE, PsycINFO, EMBASE, CINAHL, and CENTRAL. Keywords and inclusion criteria from recent related reviews were examined [15,34,47,48] and a medical librarian was consulted to verify the search strategy (eg, keywords, choice of databases). Abstract and full-text screening were independently performed by two reviewers (LE, KB, or DW), with discrepancies resolved through discussion. Multiple outputs from the same dataset were linked for included studies and imported into Endnote X7.

Included studies were peer-reviewed English-language journal articles on telehealth RCTs recruiting adult (≥18 years) type 2 diabetes patients from Western countries where English is both an official and the majority language (ie, Australia, Canada, Ireland, New Zealand, United Kingdom, United States). All other research designs and publication types were excluded, as were RCTs that included type 1 or gestational diabetes patients, or that had explicitly targeted one or more ethnic minority groups in their recruitment strategy. Interventions could comprise any telehealth medium designed to treat or improve type 2 diabetes as the primary condition. Studies focusing on secondary diabetes-related complications (eg, retinopathy) or mental health were excluded as were telehealth interventions solely targeting health professionals rather than patients.

### Data Collection

The data extraction form Multimedia Appendix 1 was developed using Cochrane guidelines [44] to describe study details, participant demographics, and intervention characteristics. Outcome data were extracted using Microsoft Excel and independently checked by two reviewers (DH, DW). The Cochrane Collaboration’s risk of bias (ROB) tool [49] was adapted to assess all 79 included articles using Cochrane’s Review Manager software (RevMan, The Cochrane Collaboration, Copenhagen, Denmark). Two of three authors (LE, DH, DW) independently evaluated each article for low, unclear, or high ROB, with discrepancies resolved through discussion. The assessment criteria were random sequence generation (selection bias), allocation concealment (selection bias), blinding of participants and personnel (performance bias), blinding of outcome assessment (detection bias), incomplete outcome data (attrition bias), selective reporting (reporting bias), and other sources of bias (other bias).

Due to the nature of telehealth interventions, patient and personnel blinding is generally unfeasible. Hence, for the purpose of this review, high risk of performance bias was interpreted as situations where unblinded research personnel interacted with participants across study groups, allowing for differential treatment. Assessment of detection bias focused on primary outcome detection. Studies that used an objective primary outcome measure (eg, laboratory-based blood test, administrative record of number of hospital visits) were assessed as low ROB because knowledge of participants’ allocation is unlikely to seriously affect the outcome. Conversely, studies where the primary outcome was subjectively assessed (eg, self-report measures) were deemed high ROB.

### Data Analysis

Data analysis of included studies addressed the following primary outcomes:

1. Proportion of studies that report on the ethnic composition of recruited participants and, where available, the overall prevalence of ethnic minority patients (between-study median and range);

2. Characteristics of studies that recruit a high proportion (≥30%) of ethnic minority participants (eg, telehealth medium, access to translation);

3. Proportion of studies that include English language proficiency or reading and writing literacy as a participant-screening criterion and, where available, the ways in which proficiency/print literacy is operationalized as a screening criterion; and

4. Language-related reasons for patient exclusion, if given (eg, informed consent, lack of resources).

In line with categorizations of race and ethnicity in the United States census [50] and conceptualizations of nonwhite or non-Caucasian respondents in other majority English-speaking countries (eg, United Kingdom [51]), ethnic minorities were defined as those of nonwhite ethnicity, including Hispanics, who may or may not be newcomers to the host country. In the case of studies reporting only the proportion of white patients recruited, all other participants were assumed to be ethnic minorities. In other studies that listed the proportion of participants belonging to an “other” group, “other” was interpreted as patients from an ethnic minority background not specified elsewhere in the study.

A narrative synthesis [52] was conducted to examine the characteristics of RCTs that reported a 30% or greater threshold of ethnic minority participants as a proportion of the total sample (considered relatively high), which is in line with the median prevalence of minority recruitment reported in earlier related reviews [3,33,34]. We would also note that this threshold is close to the proportion of ethnic minorities in the United States population. In the 2015 census, the “white alone, not Hispanic or Latino” category was reported at 61.6%, which implies that the remaining 38.4% are ethnic minorities [53]. After systematically extracting and tabulating the data, groupings and textual descriptions were used to explore heterogeneity within those studies and between studies with higher and lower proportions of ethnic minority participants. Exploration of relationships in the data was iteratively conducted to reveal factors that may promote or impede ethnic minority recruitment.

Finally, a post hoc subgroup analysis was conducted for studies that had explicitly targeted one or more ethnic minority groups as part of their recruitment strategy and that had been excluded from the systematic review for that reason only [54-65]. This additional retrospective analysis of ethnically targeted studies was undertaken to further examine recruitment strategies and features of trials that specifically recruited ethnic minority participants. This analysis involved the same methods as the synthesis described previously, but did not contribute to calculations reported in the main analysis.

## Results

### Study Selection

**Figure 1:**
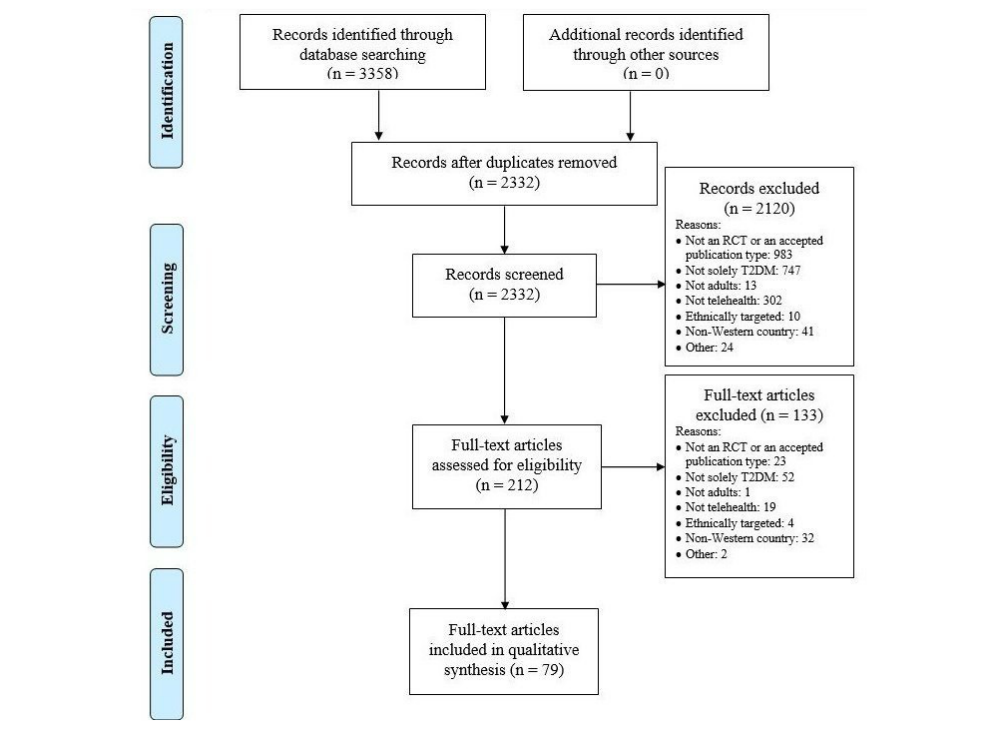
Preferred Reporting Items for Systematic Reviews and Meta-Analyses (PRISMA) flow diagram summarizing the process of selecting eligible studies for the systematic review. RCT: randomized controlled trial, T2DM: type 2 diabetes.

The search yielded 2332 records after removing duplicates, which were submitted to abstract screening. After assessing 212 full-length articles for eligibility, 79 articles, consisting of 58 discrete RCTs, met the inclusion criteria (see Figure 1).

### Characteristics of Included Studies

Multimedia Appendix 2 summarizes the characteristics of included studies in the review. The total number of participants across included RCTs was 12,916, with sample sizes ranging from 14 to 1665 (median 160). Nearly three-quarters (43/58) were recruited from the United States, whereas the rest were carried out in Australia (5/58), Canada (5/58), the United Kingdom (4/58), and one in both the United States and Canada. The studies involved a wide range of telehealth media and devices, the most popular of which was phone-based telehealth interventions. Other frequently used telehealth tools included Internet technologies (eg, static and interactive webpages, email, instant messaging), computerized self-management programs, and glucose meters integrated with mobile apps. Several studies combined different media and communication types as part of the intervention, including electronic medical records, educational websites, home monitoring, and videoconferencing (eg, [66]).

### Methodological Quality

Parts A and B of Multimedia Appendix 3 show the ROB summary table and graph for included studies. Of the 79 articles, 34 described an appropriate randomization procedure, two [67,68] reported inadequate randomization, and the remainder provided insufficient detail for assessing risk of selection bias (judged unclear). Similarly, allocation concealment reporting was frequently insufficient to determine the ROB (eg, not clear whether allocation envelopes were opaque and sequentially numbered [69]). Most studies reported designs intended to reduce performance bias (within the parameters of unfeasible patient and interventionist blinding), with studies judged as having a high ROB in cases when nonblinded staff delivered both the telehealth intervention and usual care [69-83] and also collected follow-up measures [84,85]. Included studies employed both subjective and objective outcome measures, with objective glycated hemoglobin (HbA_1c_) levels being the most common primary outcome. Approximately half of the studies included patient attrition information, with unclear ROB assigned when reasons for attrition were not given, attrition was not broken down by group allocation, or it was unclear how substantial missing data were dealt with in data analysis. Several studies were assessed as having a high ROB due to a large difference in attrition between study arms that was attributed to the intervention [86,87] or that had only presented outcomes for patients who had completed the intervention and/or all follow-up measures [88-91]. There was generally a low risk of reporting bias, with most studies reporting on prespecified outcomes. A high ROB was noted in studies where reported outcomes deviated from the protocol or specified methodologies [70,85,91-97] or insufficiently reported statistical or summary data [67,68,71,72,83,98-105]. Most studies were free of other sources of bias. However, some failed to report treatment dosage (eg, frequency of calls [74,90,106,107]) and its effect on outcomes even when adherence was low. Others were pilot studies or had small convenience samples, limiting their generalizability [71,85,95,101,108,109].

#### Ethnic Minority Participation

**Figure 2:**
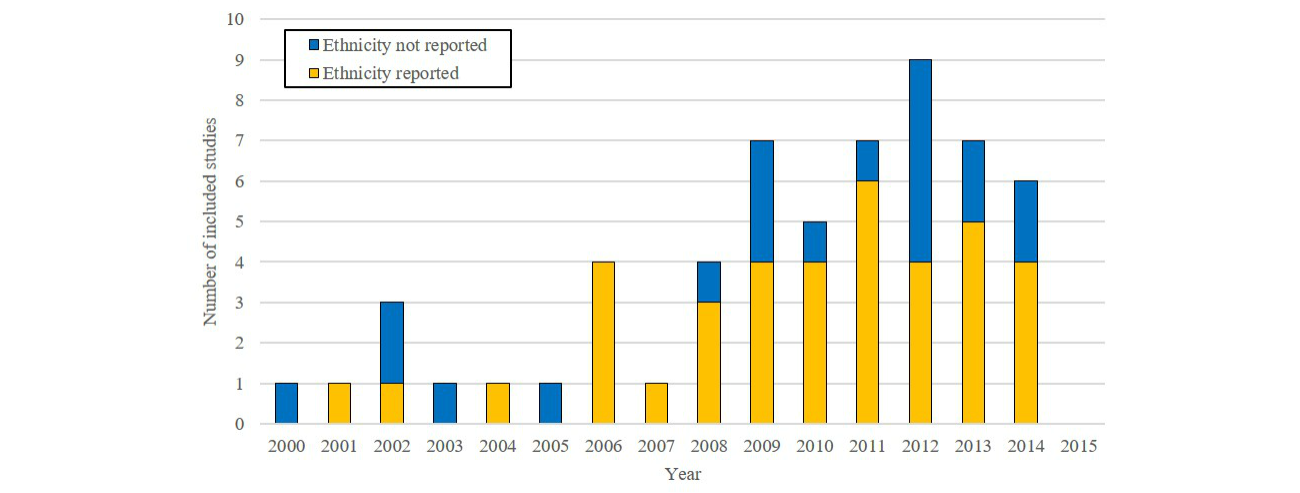
Number of included studies (n=58) reporting on the ethnic composition of the recruited sample by year of publication.

Thirty-eight of the 58 included RCTs (or 56 of the 79 articles) provided information on the ethnic composition of the sample [66-78,80,85,87,89,91-98,100-130], of which the median proportion of ethnic minority participants was 23.5% (range 0%-97.7%). Two of these recruited no ethnic minority patients [71,77]. The remaining studies (n *=* 20) provided no ethnicity information, including all five Canadian studies [79,81-84,86,88,90,99,131-144]. Figure 2 shows that the number of included studies in the review markedly increased after 2005, with eight up until that year and 50 thereafter. The proportion of the studies that reported on the ethnicity of recruited patients was 38% (3/8) up to and including 2005 and more than doubled to 70% (35/50) after that date.

#### Language Proficiency

Half of the included RCTs (29/58) reported English language proficiency as a patient inclusion or exclusion criterion, with six of these studies alternatively requiring proficiency in Spanish [66,94,105,106,110,111] and one in either Spanish or Cantonese instead of English [118]. In the 29 remaining RCTs, language ability may have been considered in recruitment but not reported in the published article, including one study that did not list any screening criteria at all [72]. Alternatively, language might not have been taken into account in recruitment. Although being able to engage with the intervention may be an implicit reason for including language as an eligibility criterion, only three studies provided explicit explanations for excluding prospective participants on language grounds. In two, this pertained to understanding study information and providing informed consent [99,109]. In the third, this related to language demands required for the intervention, which involved patients receiving tailored feedback through an automated interactive phone service [142].

Of the studies that included language proficiency as an eligibility criterion, there was little consistency in the way that this was defined. More than a third (11/29) emphasized being able to communicate in or fluently speak (and in two cases also understand) English, whereas another specified language without reference to the written medium. Of these, two studies further specified that the context for this was over the phone [91,142], which is more difficult than face-to-face communication [42]. Four other studies referred to participants needing to be able to read and speak English, seven required reading and writing, and two referred to reading and understanding (ie, receptive skills), placing no apparent emphasis on speaking or writing. Finally, five studies emphasized having English (or Spanish) as a main or primary language, implying that membership to the target language community (ie, native speaker status) was the key criterion.

From these descriptions, there were no indications that any objective measures (eg, temporal measures of fluency or oral comprehension questions) were used to establish whether prospective participants had the necessary skills to meet the specified language criterion for inclusion in the study. Reference to commonly used benchmarks of language proficiency or defined levels (eg, Canadian Language Benchmarks, Common European Framework of Reference for Languages) were not given [145]. The grounds on which a patient was determined to be linguistically eligible to participate were also unspecified. Two studies administered previously validated health literacy instruments to patients [75,118]. However, this was used to assess study outcomes in relation to health literacy and there was no threshold health literacy level required for participation. In sum, there were no explicit or standardized measures of language proficiency across included studies—a variable which could directly affect the ethnic minority patient participation [146].

#### Narrative Synthesis

Of the 79 articles reporting on 14 distinct RCTs, 28 recruited a high proportion of ethnic minority patients, with the threshold for this set at 30% or greater (median 53.3%, range 30.0%-97.7%; see Table 1) [66,70,75,85, 87,94,103,105,106, 110,111,116, 118,119]. They all took place in the United States, mostly in urban settings, with only one study exclusively recruiting from a rural setting [70]. These studies frequently recruited in medically underserved and financially deprived areas [66,70,106,110,111] and described their target populations as predominantly uninsured [75,106,111,118]. In addition to including ethnic minorities, these studies also had high numbers of patients with no insurance or who received government-sponsored Medicare or Medicaid benefits. For example, of the three studies recruiting patients from safety-net clinics, which treat uninsured patients, more than 89% were nonwhite [75,110,118]. Two studies required health care coverage as a patient screening criterion [87,103] and one solely recruited low paid health care worker union members insured through their employer [105].

**Table 1:** Summary of characteristics of 14 parent studies with a high proportion (≥30%) of ethnic minority patients.

Study	Intervention	Setting and patient characteristics^a^			Benefits of participation^b^		Tailoring of intervention^c^		
	Telehealth medium	Recruitment	Urban only	Underserved	Financial	Device	Bilingual	Cultural	Literacy
Anderson et al [106]	Phone	PC	Yes	Yes	Yes		Yes		Yes
Arora et al [110]	Mobile SMS	SN	Yes	Yes	Yes		Yes	Yes	Yes
Davis et al [70]	Videoconference & pedometer	PC	Rural	Yes	Yes			Yes	Yes
Frosch et al [111]	Video & phone	PC Com	Yes	Yes			Yes		
Glasgow et al [94]	Website & phone (automated & from an interventionist)	PC	Yes				Yes	Yes	
Khan et al [75]	Computer multimedia education	SN	Yes	Yes			Yes	Yes	Yes
Krein et al [116]	Phone	PC	Yes						
Quinn et al [85]	Bluetooth blood glucose meter with mobile app	PC Com	Mixed			Yes			
Quinn et al [87]	Mobile SMS, patient portal & phone	PC	Mixed			Yes			
Schillinger et al [118]	Phone	SN	Yes	Yes	Yes		Yes		Yes
Sevick et al [119]	Personal digital assistant	Com	Yes		Yes	Yes			
Shea et al [66]	Website, videoconference, electronic records & email	PC	Mixed	Yes		Yes	Yes		Yes
Tang et al [103]	Bluetooth blood glucose meter & electronic records	PC	Yes			Yes			
Walker et al [105]	Phone	Union	Yes	Yes			Yes		

^a^ Recruitment: Description of setting from which participants were recruited; PC: primary care; SN: safety net; Com: community center; Union: health care workers’ union. Urban only: refers to recruitment solely from urban areas. All mixed studies recruited from both urban and rural areas, with the exception of [85], which recruited from urban and suburban areas. Underserved: medically underserved/uninsured/low-wage patients or Medicare/Medicaid beneficiaries.

^b^ Financial: monetary incentives for research participation, including money, gift cards, or vouchers. Device: provision of technology or equipment including self-monitoring devices used in the intervention.

^c^ Bilingual: recruitment or educational materials translated into a minority language or availability of bilingual interventionists. Cultural: materials crafted for or through consultation with members from certain minority communities independent of language. Literacy: materials purposefully written at a particular grade level or staff trained to communicate with low-literacy patients.

High minority-recruiting studies used a mix of telehealth delivery modes, reflecting the breadth of technology used in the wider sample of studies included in the review. This suggests that the medium through which a telehealth intervention is delivered has little bearing on ethnic minority recruitment. In total, 36% (5/14) of the studies offered financial compensation for trial participation, with money or vouchers ranging from US $15 to US $175 per participant [70,106,110,118,119]. By comparison, only 21% (5/24) of low-recruiting studies (<30% ethnic minorities) offered financial incentives. A proportionately higher number of high minority-recruiting trials (35.7% vs 21% in low-recruiting studies) also offered patients equipment to facilitate self-monitoring, such as glucose meters and/or blood testing strips, which can impose a substantial cost to patients not covered by insurance [70,106,110,118,119], or free mobile phones if they were being used as part of the intervention [85,87,103].

Another key characteristic of high minority-recruiting studies was an emphasis on languages other than English. More than half (8/14) offered the intervention in a language other than English, including the use of bilingual staff for phone interventions [66,75,94,105,106,110,111,118], and two additionally reported using bilingual recruitment staff or translated study information to facilitate the recruitment of nonnative-English-speaking patients [94,118]. Conversely, no study with less than 30% ethnic minority participants offered bilingual interventions (see Multimedia Appendix 2). Four studies reported tailoring intervention materials to the needs and interests of African American and/or Latino patients, such as the use of video testimonials from community members [70,75,94,110], and nearly half reported writing study materials to facilitate low-literacy patients’ comprehension [66,70,75,106,110,118].

### Post Hoc Subgroup Analysis

A post hoc subgroup analysis was conducted for 12 articles comprising 11 RCTs that had been excluded during full-text screening [54-65]. The findings echoed those of the narrative synthesis (see Table 2). All ethnically targeted studies took place in urban American settings, with nearly half (5/11) reportedly recruiting from economically deprived or medically underserved areas [56,57,60,61,65]. More than half (6/11) used phone calls as part of the intervention, although other telehealth media were also used. Although only one study offered patients intervention-related equipment (laptop and telehealth peripherals) [57], six offered financial remuneration ranging from US $40 to US $60 cash or vouchers [55,59,61-63,65], with one trial additionally offering some patients up to US $200 for reducing their HbA_1c_ levels by a prespecified amount [62]. The ethnically targeted studies frequently reported tailoring interventions to their target demographic, including offering written and spoken aspects of the intervention in a language other than English [56,61,64,65], designing interventions through consultation and feedback from minority groups [55-58,60,65], and using interventionists drawn from the target cultural communities [55,61,64,65]. This latter point included building input from minorities into the recruitment strategy and/or piloting materials and procedures with them.

**Table 2:** Summary of characteristics of 11 ethnically targeted studies subjected to a post hoc subgroup analysis.

Study	Intervention	Setting and patient characteristics^a^			Incentives^b^		Tailoring of intervention^c^	
	Telehealth medium	Recruitment	Recruited group(s)	Underserved	Financial	Bilingual	Cultural	Literacy
Amoako & Skelly [55]	Phone	PC com	Older African American women		Yes		Yes	Yes
Calderón et al [56]	Video	PC	Spanish speakers	Yes		Yes	Yes	Yes
Carter et al [57]	Laptop with home monitoring, videoconference & electronic records	PC	African Americans	Yes			Yes	
Crowley et al [58]	Phone	PC	African Americans				Yes	Yes
Forjuoh et al [59]; Adepoju et al [54]^d^	Personal digital assistant	PC	African Americans & Hispanics		Yes			
Gary et al [60]	Phone	PC	African Americans	Yes			Yes	
Heisler et al [61]	Tablet computer	Com	African Americans & Hispanics	Yes	Yes	Yes	Yes	Yes
Long et al [62]	Phone	PC	African American veterans		Yes			
Lorig et al [64]	Phone	Com	Spanish speakers			Yes	Yes	
Lorig et al [63]	Website	Web	American Indians/ Alaska Natives		Yes			
Ruggiero et al [65]	Phone	PC	African Americans & Hispanics	Yes	Yes	Yes	Yes	Yes

^a^ Recruitment: Description of setting from which participants were recruited; PC: primary care; com: community center; Web: website. Recruited group(s): ethnicities targeted in recruitment and age group, gender, or profession of targeted participants where specified. Underserved: medically underserved/uninsured/low-wage patients or Medicare/Medicaid beneficiaries.

^b^ Financial: monetary incentives for research participation, including money or vouchers. Bilingual: recruitment or educational materials translated into a minority language or availability of bilingual interventionists.

^c^ Cultural: materials crafted for or through consultation with members from certain minority communities independent of language, or staff undertook cultural sensitivity training. Literacy: materials purposefully written at a particular grade level or staff trained to communicate with low-literacy patients.

^d^ These linked articles reported on the same RCT and it was unclear which was the parent study.

## Discussion

### Principal Findings

This systematic review investigated research reporting practices and prevalence estimates of ethnic minority participation in telehealth diabetes RCTs, extending previous reviews by including a broader range of telehealth technologies targeting type 2 diabetes. Nearly 66% of included studies reported on the ethnic composition of their samples. Although this proportion is higher than in previous reviews [3,33,34], it confirms the underreporting of ethnicity in peer-reviewed journal articles. However, compared to the 2006 review that had also excluded studies recruiting participants from a single ethnic minority background [33], these results yielded lower median participation of ethnic minority patients as a proportion of the total sample (23.5% vs 39%).

All RCTs with 30% or greater ethnic minority recruitment were US-based, mostly recruited from urban areas, and frequently described recruited patients as low-income, socially deprived, or with little or no health care coverage. Participant remuneration or free telehealth monitoring devices incentivized participation in nearly 60% of these studies. Although the United States has a high proportion of ethnic minorities compared to other countries included in the review [147], its dominance as the setting for all studies meeting the 30% or greater cut-off for the narrative review and for all ethnically targeted studies in the post hoc subgroup analysis suggests that the American National Institutes of Health (NIH) *Policy and Guidelines on the Inclusion of Women and Minorities as Subjects in Clinical Research* [148] has been influential in promoting minority representation in trials. Overall, minority participation rates tend to be higher in the United States, which has legislation to mandate their inclusion, than in contexts where no such legislation exists (eg, United Kingdom) [26]. The availability of trial materials in multiple languages was also a recurrent feature of studies recruiting substantial numbers of ethnic minority participants and often occurred in conjunction with trial materials that accounted for cultural factors or presumed literacy levels. This suggests that having a language concordant interventionist and embedding input from members of target communities into trial materials could have positive effects on the recruitment of groups who may otherwise be underrepresented [149,150]. This supports existing research on strategies to optimize the recruitment of underserved patients, which reveals ways of working holistically to alleviate individual and external factors that impede participation [146,151]. The emphasis on patient and public involvement in research aligns with such practices [29,152].

Half of the included studies listed language proficiency among the patient eligibility criteria, although less than 5% provided a reason for including or excluding patients on this basis. The role of language in participant screening was described in different ways across studies, with emphasis either placed on different combinations of skills (speaking, listening, reading, writing) to reflect the nature of the intervention, or on patients’ status as primary speakers of English or another language where offered. There was no evidence of the use of objective measures or instruments or of a common procedure across studies to assess whether patients had the requisite language proficiency to participate and, in individual studies, this level of detail was not given. Thus, it was unclear how language-related determinations about inclusion or exclusion were made—that is, how the language-screening criterion as stated was operationalized in arriving at eligibility decisions.

### Limitations

This review has several limitations. First, only English-language articles that recruited patients from Western countries where English is both an official and the dominant language were considered. Findings may be different in reviews focusing on recruitment from other contexts or on other world languages. Second, ethnic minorities are not a monolith, and language barriers to engaging with the intervention are likely to be different for newcomers to a country (eg, migrants), who may have little knowledge of English, than for later generations of native English speakers who are visible minorities (eg, African Americans in the United States) [24]. It is often difficult to disentangle ethnicity from language in secondary data analysis because the language background of participants (ie, proportion of native English speakers and heritage language speakers) is often not reported as a separate category and, in some countries, is not captured in census data [40]. Third, and related to this, we used the ethnicity categories reported in the original studies in data extraction. However, race and ethnicity are complex constructs that frequently conflate social identity with other factors, such as genetic or biological characteristics (particularly as connoted in the former term), geographical origin, cultural practices, or religious persuasion [31]. Categorizations were inconsistent across studies included in this review, making comparisons between and within countries difficult. Fourth, the prevalence of minority patients in the area(s) from which they were recruited was not possible to collate, with several studies recruiting from different geographic sites and not reporting on local population or, in some cases, on the ethnic breakdown of their recruited sample. Finally, peer-reviewed articles were the sole publication type considered in this study, which was consistent with the goal of elucidating research reporting practices in academic journals. That is, the grey literature was not examined and study authors were not contacted to provide further information than was included in their published articles, taking into account linked publications. The brief descriptions of study setting, design, and recruitment in the included telehealth RCTs provided comparatively little qualitative data for the narrative synthesis. This has resulted in a relatively simple theoretical account of the trial features supporting ethnic minority participation, which would need to be corroborated through a more in-depth examination of key variables in subsequent research. Nonetheless, with 79 articles and 58 RCTs included, this review is comprehensive in its account of research reporting and includes more studies than the three previous telehealth diabetes reviews combined [3,33,34].

### Concluding Remarks

Despite the link between new technologies and improved outcomes, mixed evidence regarding reducing disparities from the research literature [122,129] suggests that telehealth has yet to fulfill its potential of being truly accessible to and effective for ethnic minority diabetes patients [15]. Cultural and linguistic tailoring to a diverse demographic and offering translation and interpretation services where possible could extend the benefits of telehealth type 2 diabetes interventions to a wider cross-section of patients, thereby promoting more equitable access to health care [43,153].

Findings from this and earlier systematic reviews suggest that between a third and half of telehealth diabetes trials provide no information on the ethnic composition of their samples. Further, other demographic characteristics, such as socioeconomic status, are not consistently reported across studies (see Multimedia Appendix 2). Providing data on participants’ gender and age, but no information on ethnicity for either the sample or the local target population is insufficient for assessing the external validity of the findings. Ethnicity information is necessary to evaluate claims about telehealth’s accessibility to all patients (and not just a subsection of the population), including its ability to foster social inclusion through the uptake of services.

The Consolidated Standards of Reporting Trials (CONSORT) statement [154], which articulates guidelines for best practice in research reporting and which has been widely adopted, advocates reporting baseline demographic and clinical characteristics for each group in the RCT. However, this is prefaced on the assumption that these will be collected, and the statement does not specify which baseline variables should be captured and described. In the case of clinical baseline variables, further specification of which variables to target would appear to be dependent on the nature of the condition being examined (eg, HbA_1c_ for diabetes). However, this is not the case for sociodemographic variables, which apply regardless of the disease type being investigated. In trials targeting chronic conditions in which prevalence is known to vary by ethnicity and other social factors, further specification of the baseline demographic characteristics that should be reported on could reduce the variability in between-study reporting. This would assist in examining crucial relationships between sociodemographic variables and health outcomes. Journal editors and editorial boards could converge on the most important sociodemographic characteristics that should be reported (eg, in a checklist) to achieve greater consistency and help streamline such information across studies.

Finally, the fact that telehealth trials seek to recruit patients with an adequate level of language ability is understandable— telehealth interventions rely on effective spoken or written communication as a key part of the “treatment.” However, the absence of an objective or standardized measure for assessing whether patients have the requisite language ability to successfully engage with the intervention suggests that such decisions are likely being made on the basis of trial recruiters’ subjective judgments. This creates a risk of selection bias, as trial recruiters might exclude ethnic minority participants based on the subjective view that they will not be able to adequately take part in the intervention, despite actually having sufficient language proficiency [43]. For example, recruiters may misjudge having a perceptible foreign accent as evidence of poor language ability in instances when this does not actually impede communication [155,156]. Conversely, including patients who do not understand the nature of the intervention due to language barriers is problematic, not least for ethical reasons [157]. Future research could focus on the development and validation of a tool to provide trial recruiters with a simple, practical means of assessing language proficiency for trial participation to minimize the possibility of patients being unfairly excluded based on arbitrary judgments.

## Appendix

Multimedia Appendix 1 Data extraction form.

Multimedia Appendix 2 Summary of included studies in the review.

Multimedia Appendix 3 A. Risk of bias assessment summary. B. Risk of bias assessment graph.

## Abbreviations

CONSORT: Consolidated Standards of Reporting Trials
HbA_1c_: glycated hemoglobin
PRISMA: Preferred Reporting Items for Systematic Reviews and Meta-Analyses
RCT: randomized controlled trial
ROB: risk of bias
