# Supplementary material for: The Inclusion of Ethnic Minority Patients and the Role of Language in Telehealth Trials for Type 2 Diabetes: A Systematic Review
Source: J Med Internet Res. 2016 Sep 26;18(9):e256. doi: 10.2196/jmir.6374 (PMC5057063; doi:10.2196/jmir.6374)
Supplement: Multimedia Appendix 1 [file jmir_v18i9e256_app1.pdf]

Multimedia appendix 1: Data extraction form

Variables	Coding notes or categories
Study ID	
Endnote #	
Author	
Year	
Ethnicity reported?	Yes/No
Aim(s) of study	
Country	
Geographical setting	E.g., urban, rural
Recruitment setting	E.g., primary care, community, secondary care, GP referral
Study design	E.g., 2-arm/ 3-arm RCT, pilot RCT, feasibility study
English language/literacy part of inclusion/exclusion criteria?	Yes/No/Multilingual (if English is one of two or more languages permitted)
Reason(s) English language required	Informed consent/ethics; Fitness of/ability to participate in the intervention; Lack of resources; Other (specify); None given
Language proficiency definition/operationalization	Note: Provide the description then categorize according to the language skill emphasized (speaking, listening, reading, writing), whether phone communication is specified, etc.
Study's inclusion criteria	
Study's exclusion criteria	
Compensation provided for participation?	Yes (elaborate)/No
Translation/interpretation included/ bilingual interventionists	Yes (elaborate)/No
Low income/underserved/Medicare beneficiaries targeted	
Telehealth medium	E.g., videoconference, internet, mobile phone/SMS, landline, telehealth device
Intervention description	
Tailoring of Intervention	E.g., to individual needs, culture
Intervention duration	
Intervention intensity/frequency	
Intervention delivered by	E.g., nurse, GP, fully automated
Comparison group	E.g., usual care
Follow-up duration	
Total sample size	
Total male sample size	
Total sample age (*M*, SD)	
Ethnicities reported (n, %)	Note: Report using the author's original categories for race/ethnicity
Socioeconomic status indicators reported	E.g., deprivation, social class, education level, income level
Baseline diabetes severity (*M*, *SD*)	Note: Record HbA1c levels or other indicator(s) given.
Intervention effective?	Yes/No (e.g., lowered HbA1c, weight loss, increased activity)
Other Notes	
