# Supplementary material for: The Inclusion of Ethnic Minority Patients and the Role of Language in Telehealth Trials for Type 2 Diabetes: A Systematic Review
Source: J Med Internet Res. 2016 Sep 26;18(9):e256. doi: 10.2196/jmir.6374 (PMC5057063; doi:10.2196/jmir.6374)
Supplement: Multimedia Appendix 2 [file jmir_v18i9e256_app2.pdf]

Multimedia appendix 2: Summary of included studies in the review[a]

Articles (first author, year)	Country	Telehealth medium	Sample size	Sex (% male)	Age	SES variables reported on	Ethnic minorities (%)	Ethnic composition of the sample (%)	Language screening criterion
Anderson 2011 [106]	US	Phone	295	42	–	Education<br>Marital status	74.6[b]	Black 9.2<br>White 26.8<br>Other (Mixed/Asian) 64.1<br><br>Central American 0.7<br>Mexican 4.7<br>Puerto Rican 63.7<br>South American 0.7<br>Other Hispanic 5.8<br>Other 25.4	English/ Spanish primary language spoken
Arora 2014 [110]	US	Mobile SMS; landline	128	36	50.7 (10.2)	–	97.7	Hispanic/Latino 87.5<br>Black 8.6<br>White 2.3<br>Asian/Pacific Islander 1.6	Speak & read English/ Spanish
Barrera 2002 [131]	US	Internet	160	47	59.3 (9.4)	–	–	–	Read & write English
Blackberry 2013 [132]	AUS	Phone	473	47	62.8 (10.5)	–	–	–	–
Dale 2009 [91]	UK	Phone	231	59	<50 years, 17.1%; 51–69 years, 53.0%; ≥70 years, 26.9%	–	3	White British 87.9<br>White Irish 2.2<br>White Other 0.9<br>Indian 1.7<br>Black Caribbean 0.9<br>Black Other 0.4<br>Missing data 6.1	Speak (communicate) English, phone
Davis 2010 [70]	US	Video-conferencing; pedometer	165	26	59.6 (9.4)	Education<br>Income	73.9	African American/Other 73.9<br>Non-Hispanic White 26.1	–
Dy 2013 [71]	US	Video-conferencing	23	30	83 (range: 65–93)	–	0	White 100	–
Dyson 2010 [133]	UK	Video	42	43	60.8 (9.6)	–	–	–	–
Eakin 2013 [92]	AUS	Phone	302	56	58.0 (8.6)	Education<br>Employment<br>Income	12.6	Caucasian 87.4	–

Faridi 2008 [134]	US	Mobile SMS	30	37	56 (9.7)	–	–	–	–
Frosch 2011 [111]	US	DVD; phone	201	51	55.5 (8.6)	Education<br>Income<br>Health insurance	80.6	African American 15.9<br>Latino 55.7<br>White 19.4<br>Asian 3.5<br>Other 5.0	English/ Spanish primary language spoken
Gibson 2012 [135]	US	Computer program (simulation)	65	63	58.5 (range: 34–70)	–	–	–	Speak (fluency) English
Glasgow 2000 [73], 2002 [74]	US	Phone	320	44	57.4 (9.4)	Education<br>Household size<br>Employment	9.8	Caucasian 90.2	–
Glasgow 2003 [136]	US	Computer	320	47	59 (9.2)	–	–	–	Read & write English
Glasgow 2006a [112]	US	Computerized self-management program; phone	335	50	61.5 (11.3)	Education<br>Income<br>Marital status	23.3	Hispanic 17.9<br>White 76.7	Read & write English
Glasgow 2006b [72]	US	Computerized self-management program; phone	217	55	61.0 (10.7)	Education<br>Income	29.9	Non-Hispanic White 70.0<br>African American 12.0<br>Hispanic 13.8<br>Other 4.1	–
Glasgow 2012 [94], 2010 [93]	US	Internet; phone	463	50	58.4 (9.2)	Education<br>Income	45.5	American Indian/Alaska Native 6.7<br>Asian 1.6<br>Black/African American 15.4<br>White 71.9<br>Latino 21.8	Read & write English/ Spanish
Grant 2008 [98]	US	Electronic medical record	244	51	56.1 (11.6)	Neighbor-hood income	11.5	Non-White 11.5	–
Graziano 2009 [113]	US	Phone; mobile phone	119	55	61.5 (8.5)	Education<br>Household size	22.7	White 77.3<br>Non-White 22.7	Speak & understand English
Holbrook 2009 [99]	CAN	Internet; web-based diabetes tracker to interface with electronic	511	51	60.7 (12.5)	–	–	–	Speak (fluency) English[c]

		medical record; phone							
Huizinga 2010 [114]	US	Phone	164	56	55.1 (10.7)	Education	21	African American 21	English primary language spoken
Hunt 2014 [95]	US	iPad	17	41	<50 years, 35%; >51 years, 65%	Education	23.7	African American 17.7<br>Other 6	Read & write English
Jennings 2014 [137]	AUS	Internet	436	52	–	–	–	–	Understand & read English
Khan 2011 [75]	US	Computer multimedia program	129	57	51.5 (11.7)	Health literacy (outcome variable)	94.6	Hispanic 26.4<br>African American 48.8<br>White 3.1<br>Asian 17.1<br>Other 2.3	Speak (fluency) English
King 2006 [76]	US	Computer program; phone	335	50	61.5 (11.3)	Education<br>Income<br>Marital status	17.8	Hispanic 17.8<br>White 76.5	Read & write English
Krein 2004 [116]	US	Phone	246	97	61 (11.5)	Education<br>Health insurance	41.5	White 58.5	Speak English
Liebreich 2009 [144]	CAN	Internet	49	41	54.1 (10.2)	–	–	–	–
McKay 2001 [108]	US/ CAN	Internet	78	47	52.3	Education<br>Employment	18	Caucasian 82	–
McKay 2002 [138]	US	Internet	160	47	59.3 (9.4)	–	–	–	Read & write English
McMahon 2012 [69]	US	Phone; internet	151	95	60.2 (10.8)	Education<br>Employment<br>Marital status	24.6	Non-Hispanic White 74.2<br>Non-Hispanic Black 12.6<br>Hispanic 9.3<br>Other 2.7<br>No response 1.3	Understand & read English
Nagrebetsky 2013 [77]	UK	Mobile phone app; bluetooth glucose meter	14	71	58 (11)	–	0	White 100	–
Odegard 2012 [139]	US	Phone	265	48	63 (13)	–	–	–	Speak English

Pacaud 2012 [84]	CAN	Phone; internet	68	47	54.2 (9.1)	–	–	–	–
Piette 2011a [96], 2011b [117]	US	Phone	339	42	56 (10.1)	Education<br>Employment<br>Income<br>Marital status	15.8	White 83.8<br>Black 8.9<br>Other 6.9	–
Piette 2013 [78]	US	Phone	244	100	62.0(6.3)	Education<br>Household size<br>Income	18	Non-Hispanic White 82<br>Hispanic 3<br>Black 9<br>Other 6	–
Pressman 2014 [79]	US	Phone; telemetry device	254	62	55.2 (9.3)	–	–	–	–
Quinn 2008 [85]	US	Mobile phone app; bluetooth glucose meter	30	35	51.04 (11.03)	–	53.3	African American 53.3<br>White 33.3	–
Quinn 2011 [86]	US	Mobile SMS; phone	163	50	52.75	Education	47.3	Non-Hispanic Black 39.3<br>Non-Hispanic White 52.8<br>Other 8.0	–
Ralston 2009 [101]	US	Internet; email	83	51	57.3	–	18.1	Non-Hispanic White 81.9	English primary language spoken
Richardson 2007 [109]	US	Pedometer; internet	35	33	52.3 (10.6)	Education<br>Employment<br>Income	23.4	White 76.4<br>Black 13.3<br>Other 10.1	Communicate English (medium unspecified)[c]
Sacco 2009 [68], 2012 [67]	US	Phone	62	49	52 (8.6)	Education	22.6	Caucasian 77.4<br>African–American 14.5<br>Hispanic 8.1	Speak & read English
Schillinger 2008 [118]	US	Phone	339	41	55.4 (11.9)	Education<br>Health insurance<br>Health literacy (outcome variable)	92.1	Asian 22.4<br>African American 19.5<br>Hispanic/Latino 47.2<br>White 8.0<br>Other/unknown 3.0	Speak English/ Spanish/ Cantonese
Sevick 2012 [119]	US	Personal digital assistant	296	32	25–34 years, 4.2%; 35–44 years,	Education<br>Employment<br>Household size<br>Marital status	30	White 70.0	–

					10.3%; 45–54 years, 28.9%; 55–64 years, 38.0%; 65–74 years, 14.4%; ≤ 75 years, 4.2%				
Shea 2006 [66]; Izquierdo 2007 [100], 2010 [115]; Luchsinger 2011 [130]; Shea 2007 [120], 2009 [107], 2013 [102]; Trief 2006 [123], 2007 [125], 2009 [124], 2013 [122]; Weinstock 2011a [128], 2011b [129]	US	Video-conferencing; remote monitoring; electronic medical record access with email; website	1665	37	70.8	Education<br>Employment<br>Health insurance<br>Household size<br>Marital status	50.6	African-American (Non-Hispanic) 14.9<br>Hispanic 35.2<br>Non-Hispanic White 49.4<br>Other 0.5	Speak (fluency) English/ Spanish
Stone 2010 [121]	US	Telemonitoring device with messaging system; phone	137	99	<45 years, 5.1%; 45–65 years, 59.1%; ≥65 years, 35.8%	Education<br>Employment<br>Marital status	26	Non-Hispanic White 74.0<br>Non-Hispanic African-American/ Black 25.0<br>Asian/Pacific Islander 1.0<br>American Indian/Alaskan Native 0	–
Stone 2012 [80]	US	Telemonitoring device with messaging system; phone	101	98	<45 years, 1%; 45–65 years, 64%; ≥65 years, 35%	Education<br>Employment<br>Marital status	25.7	Non-Hispanic White 74.3<br>African-American (Non-Hispanic) 24.8<br>Asian/Pacific Islander 1<br>American Indian/ Alaskan Native 0	–

Tang 2013 [103]	US	Electronic medical record	415	60	53.7 (10.4)	Education	38.7	White 58.8<br>Black/African-American 5.3<br>Asian 21.5<br>Native Hawaiian 1.5<br>American Indian 1.0<br>Hispanic 9.4<br>Declined to state 2.7	Speak & read English
Tildesley 2010 [81], 2011 [140]	CAN	Internet	46	63	59.4 (9.0)	–	–	–	–
Tildesley 2013 [82], Tang 2014 [86]	CAN	Internet	50	64	58.8 (9.8)	–	–	–	–
Timmerberg 2009 [88]	US	Video-conferencing	32	50	66	–	–	–	–
Varney 2014 [97]	AUS	Phone	94	68	61.5 (range: 56–66)	–	11.7	Caucasian 88.3<br>Asian/Indian 9.6<br>Afro-Caribbean 2.1	English primary language spoken
Vigersky 2012 [141]	US	Continuous glucose monitor	100	55	57.8 (10.8)	–	–	–	–
Wakefield 2011 [104], 2012 [126]	US	Phone-mediated telemonitoring device (automated responses)	302	98	68 (10)	Education	3.7	American Indian/ Alaska Native 1<br>Black/African American 2<br>Hispanic 0.7<br>White 96.4	–
Wakefield 2014 [127]	US	Telemonitoring system (blood pressure & blood glucose readings transmitted by phone or internet)	108	44	60 (range: 29–92)	Education<br>Health insurance<br>Marital status	21.3	Non-Hispanic White 87.0<br>Black/African American 20.4<br>Asian 0.9<br>Not reported 0.9	–
Walker 2011 [105], Schreck 2014 [89]	US	Phone	526	33	55.5 (7.3)	Education<br>Income<br>Employment	94.1	Black 61.6<br>Hispanic 22.6<br>White 5.9<br>Other 9.9	Speak & read English/ Spanish

Whitlock 2000 [83]	US	Telemonitoring system (audio, video using phone lines)	28	39	63	–	–	–	–
Williams 2012 [142]	AUS	Phone	120	63	57.4 (8.3)	–	–	–	Speak & understand English, phone
Young 2005 [143], Long 2005 [90]	UK	Phone	591	58	67 (range: 22–91)	–	–	–	–

[a]The 58 parent studies examined are listed first in the rows of the table. Where applicable, linked studies are listed thereafter.
[b]For this study only, non-minority ethnicity was considered to be the 'Other' ethnicity, since it did not fall into one of the listed Central and South American categories.
[c]Ability to understand study information description or complete consent form explicitly cited as a requirement for participation.
