# Supplementary material for: The Inclusion of Ethnic Minority Patients and the Role of Language in Telehealth Trials for Type 2 Diabetes: A Systematic Review
Source: J Med Internet Res. 2016 Sep 26;18(9):e256. doi: 10.2196/jmir.6374 (PMC5057063; doi:10.2196/jmir.6374)

Multimedia appendix 3A: Risk of bias assessment summary

	1	2	3	4	5	6	7
Adepoju 2014	?	?	?	+	?	+	+
Amoako 2007	?	?	?	-	+	-	?
Anderson 2010	+	?	+	?	+	+	?
Arora 2014	+	+	+	+	+	+	+
Barrera 2002	?	?	?	-	?	+	+
Blackberry 2013	+	+	+	+	+	+	-
Calederon 2014	+	+	+	-	?	+	+
Carter 2011	+	?	+	?	-	?	+
Crowley 2013	+	+	+	+	+	+	+
Dale 2009	?	+	+	-	-	-	-
Davis 2010	?	?	-	+	?	-	?
Dy 2013	?	?	-	+	?	-	-
Dyson 2010	?	+	-	-	-	+	+
Eakin 2013	+	+	+	+	+	-	?
Faridi 2008	?	?	+	-	?	?	?
Forjuoh 2014	+	?	?	+	?	-	-
Frosch 2011	+	+	+	+	+	+	?
Gary 2009	+	?	+	+	?	+	+
Gibson 2012	?	?	-	-	+	+	?
Glasgow 2000	?	?	-	-	?	+	+
Glasgow 2002	?	?	-	-	?	+	+
Glasgow 2003	?	?	+	-	?	?	+
Glasgow 2006a	+	?	-	-	+	-	+
Glasgow 2006b	?	?	?	-	+	+	?
Glasgow 2010	+	?	?	-	+	-	+
Glasgow 2012	+	?	?	-	+	-	+
Grant 2009	?	?	+	+	+	-	-
Graziano 2009	+	+	?	+	+	+	?
Heisler 2014	+	?	-	-	+	?	?
Holbrook 2009	+	?	+	+	+	-	?
Huizinga 2010	+	+	+	+	+	+	+
Hunt 2014	?	?	+	-	?	-	+
Izquierdo 2007	?	?	+	-	?	-	-
Izquierdo 2010	?	?	+	+	+	+	?
Jennings 2014	+	?	+	-	+	+	+
Khan 2011	+	+	-	?	+	+	+
King 2006	?	?	-	-	+	+	+
Krein 2004	?	?	+	+	+	+	?
Liebreich 2009	?	?	+	-	+	+	+
Long 2005	?	?	+	-	-	+	?
Long 2012	+	?	+	+	+	+	+
Lorig 2008	?	?	+	-	-	+	+
Lorig 2010	+	?	?	?	+	+	+
Luchsinger 2011	?	?	+	-	?	+	+
McKay 2001	?	+	+	-	?	+	+
McKay 2002	?	?	?	?	+	+	+
McMahon 2012	+	?	-	+	+	+	+
Nagrebetsky 2014	+	?	-	?	+	-	+

	1	2	3	4	5	6	7
Odegard 2012	+	?	?	-	+	+	+
Pacaud 2012	?	?	-	+	?	+	+
Piette 2011a	+	+	+	?	?	-	?
Piette 2011b	+	+	+	?	?	+	+
Piette 2013	+	?	-	+	?	+	+
Pressman 2014	+	+	-	+	?	-	+
Quinn 2008	?	?	-	+	+	-	-
Quinn 2011	+	?	+	+	-	+	?
Ralston 2009	+	?	+	+	+	-	?
Richardson 2007	?	?	+	+	?	+	+
Ruggiero 2014	+	?	?	-	?	+	+
Sacco 2009	-	-	+	-	+	-	+
Sacco 2012	-	-	+	+	+	-	?
Schillinger 2008	?	?	+	+	?	?	+
Sevick 2012	+	?	+	+	+	+	?
Shea 2007	?	?	+	+	?	+	+
Shea 2006	?	+	+	+	+	+	?
Shea 2009	?	?	+	+	+	+	?
Shea 2013	?	?	+	+	+	-	?
Shreck 2014	?	?	+	-	-	+	+
Stone 2010	+	+	+	+	+	+	+
Stone 2012	+	?	-	+	+	-	+
Tang 2013	+	+	+	+	+	-	+
Tang 2014	+	?	?	-	-	+	+
Tildesley 2010	+	?	-	+	?	+	+
Tildesley 2011	+	?	-	+	?	+	-
Tildesley 2013	+	?	-	+	?	+	+
Timmerberg 2009	?	?	?	?	-	+	?
Trief 2006	?	?	+	-	?	+	?
Trief 2007	?	?	+	-	?	+	+
Trief 2009	?	?	+	-	?	+	+
Trief 2013	?	?	+	-	+	+	+
Vamey 2014	+	+	+	+	?	-	+
Vigersky 2012	?	?	+	+	?	+	+
Wakefield 2011	?	+	?	+	+	-	+
Wakefield 2012	?	?	?	-	?	+	+
Wakefield 2014	?	+	?	+	-	+	?
Walker 2011	+	?	+	+	+	-	+
Weinstock 2011a	?	?	+	-	?	+	+
Weinstock 2011b	?	?	+	+	?	+	+
Whitlock 2000	?	?	-	+	+	-	+

1	Random sequence generation (selection bias)
2	Allocation concealment (selection bias)
3	Blinding of participants and personnel (performance bias)
4	Blinding of outcome assessment (detection bias)
5	Incomplete outcome data (attrition bias)
6	Selective reporting (reporting bias)
7	Other sources of bias (other bias)

(green)	Low risk of bias
(yellow)	Unclear risk of bias
(red)	High risk of bias

Multimedia appendix 3B: Risk of bias assessment graph

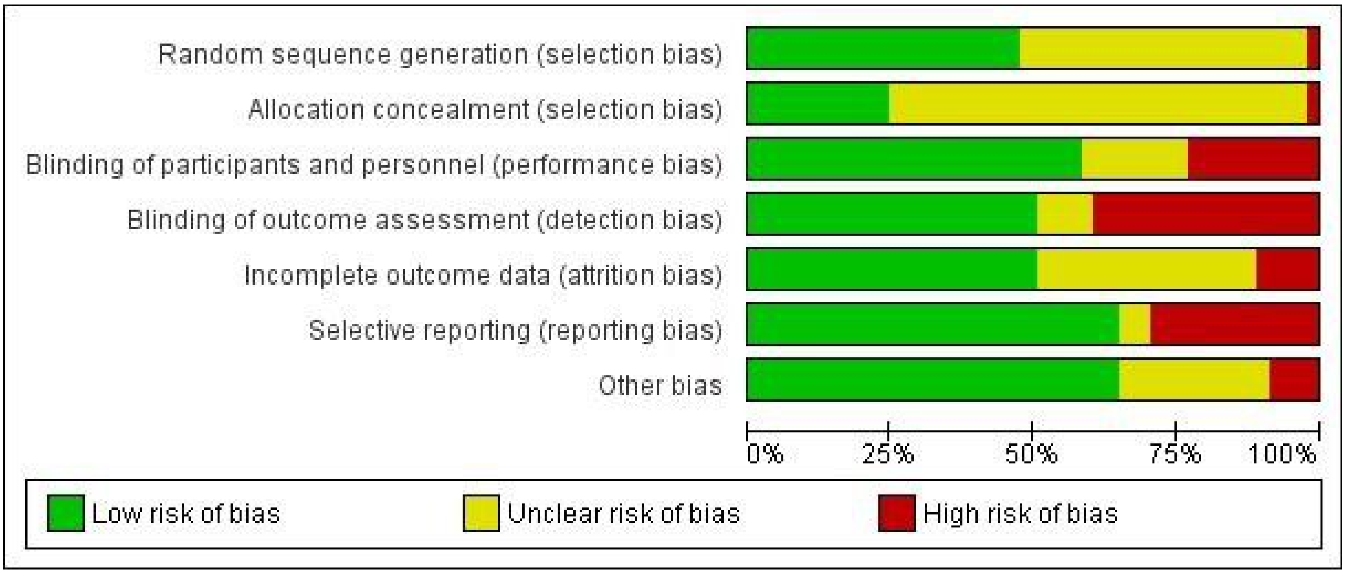

Supplement: Multimedia Appendix 3 [file jmir_v18i9e256_app3.pdf]
